# Contribution of risk factors to excess mortality in isolated and lonely individuals: an analysis of data from the UK Biobank cohort study

**DOI:** 10.1016/S2468-2667(17)30075-0

**Published:** 2017-05-04

**Authors:** Marko Elovainio, Christian Hakulinen, Laura Pulkki-Råback, Marianna Virtanen, Kim Josefsson, Markus Jokela, Jussi Vahtera, Mika Kivimäki

**Affiliations:** a Department of Psychology and Logopedics, Faculty of Medicine, University of Helsinki, Helsinki, Finland; b Helsinki Collegium for Advanced Studies, University of Helsinki, Helsinki, Finland; c Clinicum, Faculty of Medicine, University of Helsinki, Helsinki, Finland; d Department of Health Services Research, National Institute for Health and Welfare, Helsinki, Finland; e Finnish Institute of Occupational Health, Helsinki, Finland; f Department of Public Health, University of Turku and Turku University Hospital, Turku, Finland; g Department of Epidemiology and Public Health, University College London, London, UK

## Abstract

**Background:**

The associations of social isolation and loneliness with premature mortality are well known, but the risk factors linking them remain unclear. We sought to identify risk factors that might explain the increased mortality in socially isolated and lonely individuals.

**Methods:**

We used prospective follow-up data from the UK Biobank cohort study to assess self-reported isolation (a three-item scale) and loneliness (two questions). The main outcomes were all-cause and cause-specific mortality. We calculated the percentage of excess risk mediated by risk factors to assess the extent to which the associations of social isolation and loneliness with mortality were attributable to differences between isolated and lonely individuals and others in biological (body-mass index, systolic and diastolic blood pressure, and handgrip strength), behavioural (smoking, alcohol consumption, and physical activity), socioeconomic (education, neighbourhood deprivation, and household income), and psychological (depressive symptoms and cognitive capacity) risk factors.

**Findings:**

466 901 men and women (mean age at baseline 56·5 years [SD 8·1]) were included in the analyses, with a mean follow-up of 6·5 years (SD 0·8). The hazard ratio for all-cause mortality for social isolation compared with no social isolation was 1·73 (95% CI 1·65–1·82) after adjustment for age, sex, ethnic origin, and chronic disease (ie, minimally adjusted), and was 1·26 (95% CI 1·20–1·33) after further adjustment for socioeconomic factors, health-related behaviours, depressive symptoms, biological factors, cognitive performance, and self-rated health (ie, fully adjusted). The minimally adjusted hazard ratio for mortality risk related to loneliness was 1·38 (95% CI 1·30–1·47), which reduced to 0·99 (95% CI 0·93–1·06) after full adjustment for baseline risks.

**Interpretation:**

Isolated and lonely people are at increased risk of death. Health policies addressing risk factors such as adverse socioeconomic conditions, unhealthy lifestyle, and lower mental wellbeing might reduce excess mortality among the isolated and the lonely.

**Funding:**

Academy of Finland, NordForsk, and the UK Medical Research Council.

## Introduction

Socially isolated and lonely individuals have a higher mortality risk than people with social contacts.^1^, ^2^, ^3^, ^4^, ^5^ Several factors might contribute to these associations.^6^ According to one hypothesis, losing social connections and feeling lonely could be associated with depressive mood and cognitive decline,^7^ with accompanying downstream biological changes such as increased cortisol secretion, deterioration in immune function, and weight gain.^6^ Social isolation could also be associated with unhealthy lifestyle factors, such as increased smoking, increased alcohol consumption, and physical inactivity.^8^ Similarly, socioeconomic adversity is associated with an increased likelihood of social isolation,^9^ and thus might explain the reported associations. However, few extensive prospective data exist on which to test these hypotheses and assess the associations in different groups such as old and young individuals, low and high socioeconomic groups, and those with and without chronic disease. All these factors might confound the association of social isolation and loneliness with mortality.

A better understanding of the factors underlying the associations between social isolation (ie, having no or few contacts with others), loneliness (ie, feeling lonely or unable to share one's thoughts), and mortality might facilitate the design of interventions to reduce excess health risk in socially isolated, lonely people. We used data from the UK Biobank study to quantify the extent to which the associations of social isolation and loneliness with mortality are related to biological, behavioural, socioeconomic, and psychological risk factors.

## Methods

### Study design and participants

We analysed baseline data and mortality follow-up data from the UK Biobank study.^10^ UK National Health Service registers maintain records of all individuals legally registered as resident in the UK. With the help of these records, invitations were sent to individuals aged 40–69 years living within a sensible travelling distance of the 22 assessment centres across Great Britain in 2007–10.^10^ For the UK Biobank project, baseline questionnaires and physical measures (eg, standard anthropometry and spirometry) were collected and blood and urine samples were stored, as described elsewhere.^11^ 502 656 individuals were recruited (5% of the eligible population) in the UK Biobank.

**Research in context**

**Evidence before this study**

Social isolation and loneliness are associated with increased health problems and excess mortality risk. We searched PubMed for studies published in English up to May 31, 2016, using the following combinations of search terms (in title): (A) social support AND mortalit*; (B) social relations AND mortalit*; (C) social networks AND mortalit*; (D) social isolation AND mortalit*; and (E) loneliness AND mortalit*. Search combination A yielded 37 publications, B eight, C 14, D 11, and E 14. Findings from these studies suggest that there is an association between social isolation and mortality and between loneliness and mortality.

**Added value of this study**

Our study is, to our knowledge, the largest investigation into factors linking social isolation and loneliness to an increased mortality risk. We did a mediation analysis and found that the association between social isolation and mortality reduced by 64% after taking into account differences in lifestyle, socioeconomic factors, and mental health problems between socially isolated and non-isolated individuals. These risk factors explained the association between loneliness and mortality.

**Implications of all the available evidence**

Isolation and loneliness are markers of many risk factors, such as socioeconomic adversity, unhealthy lifestyles, and lowered mental wellbeing. Policies and public health interventions that tackle these risk factors in general could potentially reduce excess mortality among the isolated and the lonely.

This study was done under generic approval from the National Health Service National Research Ethics Service (June 17, 2011; Ref 11/NW/0382). Participants provided electronic informed consent for the baseline assessments and the register linkage.

### Procedures

The social isolation scale used by the UK Biobank was constructed from three questions: (1) “Including yourself, how many people are living together in your household? Include those who usually live in the house such as students living away from home during term time, partners in the armed forces or professions such as pilots” (1 point for living alone); (2) “How often do you visit friends or family or have them visit you?” (1 point for friends and family visit less than once a month); and (3) “Which of the following [leisure/social activities] do you engage in once a week or more often? You may select more than one” (1 point for no participation in social activities at least weekly). Thus, individuals could score a total of 0–3; an individual was defined as socially isolated if he or she scored 2 or 3; those who scored 0 or 1 were classified as not isolated. Similar scales have been used previously in other UK studies.^12^

Loneliness was assessed with two questions: “Do you often feel lonely?” (no=0, yes=1) and “How often are you able to confide in someone close to you?” (0=almost daily to once every few months; 1=never or almost never). An individual was defined as lonely if he or she scored 2, and not lonely if he or she scored 0 or 1. Similar questions are included in scales such as the revised UCLA Loneliness Scale.^13^

Follow-up for all deaths irrespective of cause started at inclusion in the UK Biobank study (from national death registers) and ended on Aug 14, 2015, or upon death, for all participants. The cause-specific-mortality International Classification of Diseases (ICD) codes were as follows: neoplasms (C00–D48), diseases of the circulatory system (I05–I89), and other diseases (all remaining ICD-10 codes).

Details of the assessments of participants' variables are publicly available.^14^ Briefly, participants completed several touch-screen computer-based questionnaires, and then had a face-to-face interview with a trained researcher. The information collected included basic demographics (sex and age), ethnic origin (white *vs* other), socioeconomic factors (educational attainment, household income, and postcode of residence with the corresponding Townsend deprivation index score), and chronic diseases (diabetes, cardiovascular disease, cancer, and other long-standing illness, disability, or infirmity). The Townsend deprivation index is an integrated neighbourhood-level measure of unemployment, non-car ownership, non-home ownership, and household overcrowding across the UK.^15^

To assess biological factors, trained data collectors measured height and weight in all participants during clinic attendance using standard operating procedures, and the body-mass index (BMI) was subsequently calculated. Procedures for measuring systolic and diastolic blood pressure and handgrip strength are reported in the UK Biobank protocol, which is available online.^11^ Behavioural factors, including cigarette smoking (current smoker [yes or no]; ex-smoker [yes or no]), physical activity (moderate and vigorous), and alcohol intake frequency (at least three times a week *vs* twice a week or less) were self-reported on a questionnaire.

Psychological factors comprised current depressive symptoms and general cognitive capacity. Depressive symptoms were measured using the frequency of four items from the Patient Health Questionnaire (PHQ):^16^, ^17^ (1) depressed mood, (2) disinterest or absence of enthusiasm, (3) tenseness or restlessness, and (4) tiredness or lethargy in the previous 2 weeks. General cognitive capacity (numeric memory, verbal–numerical reasoning, reaction time, and visual memory) was assessed by use of a touch-screen application.^18^ Self-rated health was assessed using the following question answered on a four-point scale (1=poor; 4=excellent): “In general, how would you rate your overall health?”

### Statistical analysis

We did analyses first using data from those participants who did not have any missing data (complete case analyses) and then using imputed datasets. We examined the associations of social isolation and loneliness with all-cause mortality using Cox proportional hazard models with age as a timescale. Associations with cause-specific mortality were examined using competing-risks survival regression (based on Fine and Gray's proportional sub-hazards model), which is the appropriate method for estimation of competing actual risks.^19^ All the models were adjusted for age, sex, and ethnic origin, with additional adjustment for chronic disease. To measure the robustness of these associations, we did additional subgroup analyses separately for men and women, three age groups (37–52 years, 53–60 years, and 61–73 years), different ethnic groups (white *vs* non-white), and participants with and without chronic disease at baseline. Subgroup analyses by sex, age, ethnic origin, and chronic disease were chosen because these factors represent potential confounders for the association between social relations and mortality. Men, individuals belonging to ethnic minorities, elderly people, and those with long-standing illness tend to have fewer social relations and also are at increased risk of mortality.^4^, ^6^ Similar three-level age categorisations have been used in a previous study based on UK Biobank data.^20^

To assess the extent to which baseline biological, behavioural, socioeconomic, psychological, and health-related risk factors explained the associations of social isolation and loneliness with mortality, we calculated the percentage of excess risk mediated (PERM) for the following five groups of explanatory variables: (1) biological (BMI, diastolic and systolic blood pressure, and handgrip strength); (2) behavioural (smoking, alcohol consumption, and physical activity); (3) socioeconomic (Townsend deprivation index, education, and household income); (4) psychological factors (depressive symptoms and cognitive performance); and (5) self-rated health. For each risk-factor group, we estimated the percentage of PERM as:^21^

$$\text{PERM}=\left[\frac{\begin{array}{l} \text{Hazard}\;\mathrm{0}\text{ratio}\;(\text{age},\;\text{sex}\;\text{ethnicity},\;\text{and}\\ \text{chronic}\;\text{disease}\;\text{adjusted})-\text{hazard}\\ \text{ratio}\;(\text{age},\;\text{sex},\;\text{ethnicity},\;\text{chronic}\\ \text{disease},\;\text{and}\;\text{risk}\;\text{factor}\;\text{adjusted}) \end{array}}{\begin{array}{l} \text{Hazard}\;\mathrm{0}\text{ratio}\;(\text{age},\;\text{sex}\;\text{ethnicity},\;\text{and}\\ \text{chronic}\;\text{disease}\;\text{adjusted})-1 \end{array}}\right]\times 100$$

Sex, ethnic origin, chronic disease, smoking status, education, and high alcohol consumption were treated as categorical and the other risk factors as continuous variables in the analyses. Finally, all the risk factors were included in the same model simultaneously (final model).

To assess the extent to which the associations followed a dose–response pattern, we did dose–response analyses using sum scores from the individual items of the isolation and loneliness measures. We analysed isolation and loneliness separately to assess whether there was a pattern across the continuous score as a predictor of mortality.

We accounted for missing data by multiple imputation by chained equations, which generated five imputed datasets.^22^ The imputation model included age, sex, social isolation, loneliness, all confounding and mediating variables, the Nelson-Aalen estimate of cumulative hazard, and survival status.^23^ We fitted Cox proportional hazards models within each imputed dataset and combined them in accordance with Rubin's rules.

To test whether reverse-causation bias (ie, the effect of chronic disease on social isolation) affected our results, we did a sensitivity analysis examining the association between social isolation and all-cause mortality (imputed data, but chronic disease missingness not imputed) after adjustment for all covariates in those without chronic disease at baseline. We used Stata (version 13.1) for all analyses.

### Role of the funding source

The funders of the study had no role in study design, data collection, data analysis, data interpretation, or writing of the report. ME and CH had full access to all the data in the study and ME and MK had final responsibility for the decision to submit for publication.

## Results

466 901 (93%) of the 502 656 individuals recruited to the UK Biobank provided complete data on social isolation, loneliness, and mortality, and were included in the present analysis. There were statistically significant differences in all the study variables between those who were and those who were not included, although the absolute between-group differences were small (appendix p 2). The mean age of study participants was 56·5 years (SD 8·1; range 40–69), 55% were women, and 95% were white (table). 42 548 (9%) of 466 901 participants were categorised as socially isolated and 29 442 (6%) as lonely.

**Table:** Descriptive statistics of the study participants

		**Participants (n=466 901)**
Age (years)		56·5 (8·1)
Sex		
	Women	254 919 (55%)
	Men	211 982 (45%)
Ethnic origin		
	Non-white	21 482 (5%)
	White	444 118 (95%)
	Data missing	1301 (<1%)
Townsend index score*		–1·37 (3·05)
Education		
	No secondary education	77 329 (17%)
	Secondary education	232 222 (50%)
	University degree	153 810 (33%)
	Data missing	3540 (1%)
Annual household income		
	Less than £31 000	193 196 (41%)
	At least £31 000	212 753 (46%)
	Data missing	60 952 (13%)
Chronic illness		
	No	224 947 (48%)
	Yes	229 595 (49%)
	Data missing	12 359 (3%)
Social isolation		
	No	424 353 (91%)
	Yes	42 548 (9%)
Loneliness		
	No	437 459 (94%)
	Yes	29 442 (6%)
Body-mass index (kg/m^2^)		27·4 (4·8)
Diastolic blood pressure (mm Hg)		82·2 (10·1)
Systolic blood pressure (mm Hg)		137·8 (18·6)
Handgrip strength (kg)		30·7 (11·0)
Smoker		
	No	416 921 (89%)
	Yes	48 542 (10%)
	Data missing	1438 (<1%)
Ex-smoker		
	No	303 113 (65%)
	Yes	162 350 (35%)
	Data missing	1438 (<1%)
Alcohol intake frequency		
	Twice or less per week	261 310 (56%)
	At least three times per week	205 351 (44%)
	Data missing	240 (<1%)
Physical activity†		
	Moderate (range 0–7)‡	3·7 (2·3)
	Vigorous (range 0–7)§	1·9 (1·9)
Cognitive performance (range 0–13)^18^		6·2 (2·1)
Patient Health Questionnaire		
	Depressed mood (range 1–4)	1·3 (0·6)
	Disinterest or no enthusiasm (range 1–4)	1·3 (0·6)
	Tenseness or restlessness (range 1–4)	1·3 (0·6)
	Tiredness or lethargy (range 1–4)	1·7 (0·8)
Self-rated health (range 1–4)		1·9 (0·7)

Data are mean (SD) or number (%). Some percentages do not add up to 100 because of rounding.

* A standardised measure of deprivation, including area-level unemployment (as a percentage of those aged 16 years and older who are economically active [ie, not retired or living in care]), non-car ownership (as a percentage of all households), non-home ownership (as a percentage of all households), and household overcrowding.

† Number of days per week of physical activity lasting more than 10 min.

‡ Activities that needed moderate effort, resulting in slight shortness of breath.

§ Activities that caused sweating or heavy breathing, such as cycling, aerobics, or heavy lifting.

During a mean follow-up of 6·5 years (SD 0·8) and 3·0 million person-years at risk, 11 593 individuals died. The most common causes of death were neoplasms (6758 deaths) and diseases of the circulatory system (2032 deaths). Other causes (2803 deaths) included diseases of the respiratory and digestive systems and external causes.

**Figure 1:**
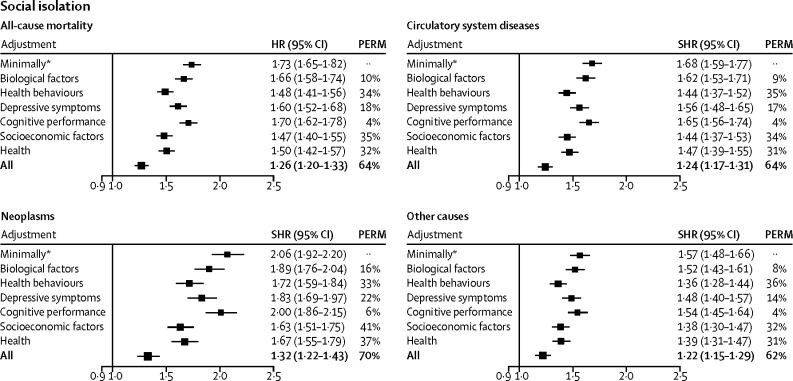
Proportions of the social isolation–mortality association attributable to biological, behavioural, and psychological factors HR=hazard ratio. PERM=percentage of excess risk mediated. SHR=sub-hazard ratio. *Adjusted for age, sex, ethnic origin, and chronic disease.

In the complete case analysis, the hazard ratio (adjusted for age, sex, ethnic origin, and chronic disease) for the risk of death from any cause among socially isolated people compared with their non-isolated counterparts was 1·74 (95% CI 1·65–1·83). This association was consistent across sex and age groups, ethnic groups, and participants with and without chronic diseases at baseline, although it was weaker in women than in men (appendix p 6). The results of the cause-specific mortality analyses followed a similar pattern to those for all-cause mortality. Socially isolated individuals had an increased risk of death from neoplasms (minimally adjusted sub-hazard ratio 2·06, 95% CI 1·92–2·20), diseases of the circulatory system (1·68, 1·59–1·77), and other causes (1·57, 1·48–1·66; figure 1).

In the multivariable analyses, the hazard ratio for social isolation compared with no social isolation was 1·73 (95% CI 1·65–1·82) after adjustment for age, sex, ethnic origin, and chronic disease (ie, minimally adjusted), and decreased by 10% after adjustment for biological risk factors, 34% after adjustment for behavioural risk factors, 35% after adjustment for socioeconomic factors, 18% after adjustment for depressive symptoms, 4% after adjustment for cognitive performance, and 32% after adjustment for self-rated health (figure 1). The overall attenuation after adjustment for all these factors (ie, fully adjusted) was 64% (hazard ratio 1·26, 95% CI 1·20–1·33). Similar patterns were found in all the cause-specific mortality groups. Socioeconomic factors (PERM 32–41%), behavioural factors (33–36%), and self-rated health (31–37%) were the strongest explanatory variables, but the fully adjusted sub-hazard ratios remained statistically significant and ranged from 1·32 to 1·22 (figure 1).

**Figure 2:**
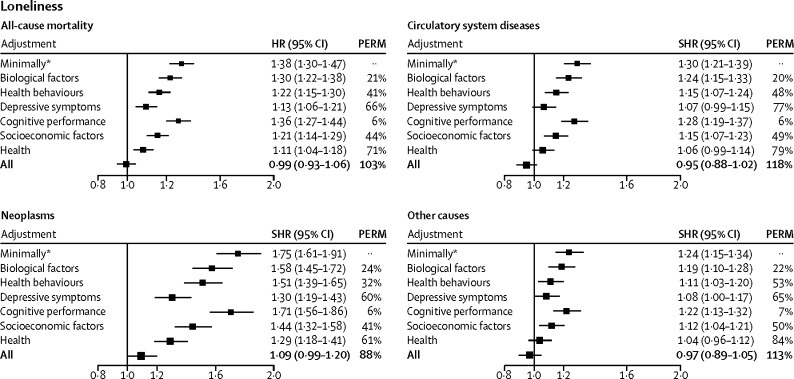
Proportions of the loneliness–mortality association attributable to biological, behavioural, and psychological factors HR=hazard ratio. PERM=percentage of excess risk mediated. SHR=sub-hazard ratio. *Adjusted for age, sex, ethnic origin, and chronic disease.

In the complete case analysis, the minimally adjusted hazard ratio for the risk of death from any cause in lonely individuals compared with those who were not lonely was 1·37 (95% CI 1·28–1·46). This association was consistent across sex and age groups, ethnic groups, and participants with and without chronic disorders at baseline (appendix p 7). Lonely individuals had an increased risk of death from neoplasms (minimally adjusted sub-hazard ratio 1·75, 95% CI 1·61–1·91), diseases of the circulatory system (1·30, 1·23–1·39), and other causes (1·24, 1·15–1·34; figure 2). Serial adjustments led to complete attenuation of the association (fully adjusted hazard ratio 0·99, 95% CI 0·93–1·06; figure 2). A similar pattern emerged in all the cause-specific mortality groups. Depressive symptoms (PERM 60–77%) and self-rated health (61–84%) seemed to be the strongest mediators, in addition to socioeconomic (41–50%) and behavioural factors (32–53%; figure 2). Social isolation and loneliness were associated with higher levels of depressive symptoms, smoking, and high alcohol intake frequency, all of which are well established correlates of social isolation and loneliness (appendix p 3).

The multivariable, minimally adjusted complete-case analysis showed similar associations between all-cause mortality and social isolation (hazard ratio 1·29, 95% CI 1·14–1·47) and loneliness (0·93, 0·77–1·11).

The dose–response analyses revealed a dose–response pattern in the associations of social isolation and loneliness with all-cause mortality (appendix p 5). When both isolation and loneliness were tested in the same model, only social isolation predicted all-cause mortality (hazard ratio 1·27, 95% CI 1·20–1·33).

In the sensitivity analysis testing for reverse-causation bias, the association between social isolation and all-cause mortality was still apparent (hazard ratio 1·22, 95% CI 1·08–1·38). The frequencies of complete and imputed variables are reported in the appendix (p 4; imputed data sample size 499 238).

## Discussion

In this UK Biobank study, social isolation was associated with increased mortality in the total cohort as well as in the subgroups of men and women, younger and older individuals, initially healthy and unhealthy people, and ethnic subgroups. We also found a similar relation for cause-specific mortality, including deaths from neoplasms and diseases of the circulatory system. Risk factors explained 64% of the association between social isolation and mortality, leaving over a third independent of socioeconomic factors, health-related behaviours, depressive symptoms, biological factors, and cognitive capacity. Loneliness was also associated with increased mortality, but, unlike social isolation, differences in risk-factor levels, especially depressive symptoms, between lonely individuals and others explained its association with all-cause and cause-specific mortality.

To our knowledge, this is the first large-scale study on the contribution of biological, behavioural, socioeconomic, and psychological risk factors to associations between social isolation and all-cause and cause-specific mortality. Our research complements findings from meta-analyses,^3^, ^4^, ^5^ which reported an association between social isolation and increased all-cause mortality. The novel aspects in our analysis include the identification of explanatory factors with greater precision than previously, and the fact that we examined not only all-cause mortality, but also deaths from neoplasms and circulatory diseases.^4^ The strength of the social isolation–mortality association broadly reflected what has been previously reported, with a hazard ratio of 1·73 in the present study compared with 1·3 in previous meta-analyses.^3^, ^4^, ^5^ This similarity was also the case for loneliness, with a hazard ratio of 1·38 in the present study compared with a minimally adjusted relative risk of 1·3 in a previous meta-analysis.^4^ However, unlike in some previous studies, our findings suggest that the association between loneliness and mortality is fully attributable to the worse risk-factor levels in the exposed group. Of the risk factors, increased depressive symptoms explained 66% and socioeconomic factors 44% of the excess mortality in lonely individuals. This result broadly confirms the less precise estimates reported in a smaller study in elderly people.^24^

Findings from previous studies^3^, ^4^ have suggested that socioeconomic factors explain part of the association between social isolation and disease. In support of these findings, we show that 35% of the relation between mortality and social isolation was attributable to education, neighbourhood deprivation, and household income (ie, socioeconomic factors). With regard to loneliness, the contribution of socioeconomic factors was 44%. Contrary to previous findings,^6^ the present results do not imply that biological factors (eg, obesity, high diastolic and systolic blood pressure, and low handgrip strength) are major contributing factors. However, because our analyses were adjusted for prevalent chronic diseases at baseline, the estimated contribution of biological risk factors did not include variation in these diseases.

The association between mortality and social isolation seemed stronger than the association between mortality and loneliness. These two factors measure different aspects of social relations and thus also have slightly different associations with health outcomes and mortality. Whereas isolation measures the scarcity of contact with other people and related health resources, loneliness is a perception of detachment associated potentially with emotional states such as depressive symptoms. People can feel lonely even if they are married or living with someone, and that feeling might be less closely related to an absence of practical support than to actual isolation.^25^

Some methodological issues should be taken into account when interpreting our findings. Studies in this specialty have typically relied on small samples that are vulnerable to chance findings. Meta-analyses of these studies are additionally limited by heterogeneity in study populations, the diversity of measures used, and differences in the levels of statistical adjustment. The UK Biobank provided an opportunity to investigate the associations of social isolation and loneliness with mortality and their links to risk factors in a large sample, substantially reducing the risk of random error. However, the response rate was low, and selection bias should be taken into account especially when making inferences about population prevalence figures. However, prevalence and incidence were not the focus of the present study. We used a multi-item assessment of social isolation, which is referred to in previous studies as the best predictive validity for such a measurement strategy in relation to mortality.^4^ Assessing more than one type of social-relationship measurement (structural and functional) might better capture the many effects of social relationships.^5^, ^26^

We measured only simple forms of the complex phenomenon of social networks and interaction, although similar results have been reported in studies using more advanced network analysis.^27^ Missing data on the covariates reduced the sample size at each successive stage of the regression model adjustment, although the results were similar when the analyses were repeated with only participants who provided complete data on all the variables. The possibility of residual confounding cannot be completely ruled out in observational studies such as ours, although the association between social isolation and mortality remained even after adjustment for a wide range of potential confounders. Similarly, reverse causality can affect the results of observational research. For example, in our study, chronic disease might have affected the risk of both social isolation and mortality. However, we noted an association between social isolation and mortality even among those with no prevalent chronic disease at baseline and after adjustment for a range of health-related covariates. Finally, the sample comprised participants aged between 40 years and 69 years; hence, the findings cannot be extrapolated beyond this age range.

In conclusion, data from the UK Biobank suggest that social isolation is associated with overall excess mortality and death attributable to neoplasms and circulatory diseases. Most of the excess mortality among socially isolated and lonely people could be attributed to adverse socioeconomic conditions, an unhealthy lifestyle, and lower mental wellbeing. Public health policies addressing these issues might reduce this excess. Such policies have been designed to increase longevity in the general population. The results of the present study suggest that isolated and lonely people in particular would benefit from successful implementation of targeted policies. Future studies should assess the potential benefits, harms, and cost-effectiveness of interventions and policies aimed at tackling risk factors in lonely and isolated people.
