# Correction to *Lancet Public Health* 2017; 2: e260–66

**DOI:** 10.1016/S2468-2667(19)30086-6

**Published:** 2019-05-30

*Elovainio M, Hakulinen C, Pulkki-Råback L, et al. Contribution of risk factors to excess mortality in isolated and lonely individuals: an analysis of data from the UK Biobank cohort study.* Lancet Public Health *2017;* 2: *e260–66—*In the second paragraph of the Procedures section on page e261, the definitions for 0 and 1 for the question “How often are you able to confide in someone close to you?” have been corrected. These corrections have been made to the online version as of May 30, 2019.

