# Supplementary material for: Contribution of risk factors to excess mortality in isolated and lonely individuals: an analysis of data from the UK Biobank cohort study
Source: Lancet Public Health. 2017 May 4;2(6):e260–6. doi: 10.1016/S2468-2667(17)30075-0 (PMC5463031; doi:10.1016/S2468-2667(17)30075-0)
Supplement: Supplementary appendix [file mmc1.pdf]

THE LANCET
Public Health

Supplementary appendix

This appendix formed part of the original submission and has been peer reviewed. We post it as supplied by the authors.

Supplement to: Elovainio M, Hakulinen C, Pulkki-Råback L, et al. Contribution of risk factors to excess mortality in isolated and lonely individuals: an analysis of data from the UK Biobank cohort study. *Lancet Public Health* 2017; published online May 4. http://dx.doi.org/10.1016/S2468-2667(17)30075-0.

Online Supplement Appendix

Contents

1. Supplement Tables 1-4
2. Supplement Figures 1-2

Supplement Table 1. Differences between excluded and included participants

Variable	Excluded<br>Mean or percentage	Included<br>Mean or percentage
Age (years)	56.76	56.51
Male	48.10 %	45.40 %
Caucasian	83.78 %	95.39 %
Deprivation index	-0.32	-1.37
Education (university)	25.39 %	33.19 %
Annual household (income 31k or greater)	37.25 %	52.41 %
Chronic illness (yes)	55.11 %	50.51 %
BMI (kg/m^2)	27.67	27.42
Diastolic BP (mm Hg)	82.43	82.2
Systolic BP (mm Hg)	138.54	137.77
Handgrip strength (kg)	29.34	30.7
Smoker	12.99 %	10.43 %
Ex-smoker	31.41 %	34.88 %
Alcohol intake frequency	0.35	0.44
Moderate physical	3.71	3.62
Vigorous physical	1.82	1.84
Cognitive performance	5.18	6.03
Depressed mood	1.38	1.3
Unenthusiasm / disinterest	1.4	1.28
Tenseness / restlessness	1.4	1.33
Tiredness / lethargy	1.84	1.72
Self-rated health	1.69	1.87

Note. Values are means or percentages. All differences are statistically significant ($p<.05$).

Supplemental Table 2. Pairwise correlation coefficients between social isolation, loneliness, and potential confounders

	1.	2.	3.	4.	5.	6.	7.	8.	9.	10.	11.	12.	13.	14.	15.	16.	17.	18.	19.
1. Social isolation	100	13	-5	-13	3	1	0	-2	9	-1	-7	-4	-7	-5	10	10	7	8	-11
2. Loneliness	13	100	-6	-8	5	0	-2	-2	6	-1	-5	-2	-2	-4	27	24	20	17	-14
3. Education	-5	-6	100	35	-14	-4	-13	11	-8	-6	14	-6	1	37	-5	-7	-4	-6	18
4. Annual household	-13	-8	35	100	-8	-1	-11	18	-8	-3	16	-9	2	24	-9	-10	-6	-7	18
5. BMI (kg/m2)	3	5	-14	-8	100	28	19	4	-3	8	-11	-10	-9	-6	6	8	3	12	-27
6. Diastolic BP (mm Hg)	1	0	-4	-1	28	100	70	16	-2	2	6	-2	-2	-2	-2	0	-2	-2	-7
7. Systolic BP (mm Hg)	0	-2	-13	-11	19	70	100	9	-5	6	7	3	1	-4	-8	-5	-7	-9	-5
8. Handgrip strength (kg)	-2	-2	11	18	4	16	9	100	4	4	15	2	13	11	-8	-3	-6	-13	9
9. Smoker	9	6	-8	-8	-3	-2	-5	4	100	-25	1	0	-3	-6	9	11	8	8	-13
10. Ex-smoker	-1	-1	-6	-3	8	2	6	4	-25	100	13	1	1	2	-2	-2	-1	-2	-3
11. Alcohol intake frequency	-7	-5	14	16	-11	6	7	15	1	13	100	0	2	16	-6	-7	-4	-10	12
12. Moderate physical	-4	-2	-6	-9	-10	-2	3	2	0	1	0	100	49	-10	-3	-4	-2	-8	11
13. Vigorous physical	-7	-2	1	2	-9	-2	1	13	-3	1	2	49	100	-7	-4	-4	-3	-11	18
14. Cognitive performance	-5	-4	37	24	-6	-2	-4	11	-6	2	16	-10	-7	100	-9	-12	-9	-7	13
15. Depressed mood	10	27	-5	-9	6	-2	-8	-8	9	-2	-6	-3	-4	-9	100	62	57	43	-27
16. Unenthusiasm / disinterest	10	24	-7	-10	8	0	-5	-3	11	-2	-7	-4	-4	-12	62	100	49	42	-27
17. Tenseness / restlessness	7	20	-4	-6	3	-2	-7	-6	8	-1	-4	-2	-3	-9	57	49	100	42	-24
18. Tiredness / lethargy	8	17	-6	-7	12	-2	-9	-13	8	-2	-10	-8	-11	-7	43	42	42	100	-39
19. Self-rated health	-11	-14	18	18	-27	-7	-5	9	-13	-3	12	11	18	13	-27	-27	-24	-39	100

Note. Correlation coefficients are multiplied by 100.

Supplement Table 3. Frequencies of complete and imputed variables

Variable	Complete	Imputed	Total
BMI (kg/m^2)	496566	2672	499238
Diastolic BP (mm Hg)	469637	29601	499238
Systolic BP (mm Hg)	469632	29606	499238
Handgrip strength (kg)	497277	1961	499238
Moderate physical (range 0-7)	473193	26045	499238
Vigorous physical (range 0-7)	472892	26346	499238
Education	490210	9028	499238
Annual household income	423762	75476	499238
Depressed mood	476325	22913	499238
Unenthusiasm / disinterest	480687	18551	499238
Tenseness / restlessness	478324	20914	499238
Tiredness / lethargy	483188	16050	499238
Cognitive performance	164571	334667	499238
Social isolation	487847	11391	499238
Loneliness	474325	24913	499238
Smoker	497328	1910	499238
Ex-Smoker	497328	1910	499238
Alcohol intake frequency	498767	471	499238
Chronic illness	483744	15494	499238
Self-rated health	496842	2396	499238

Supplemental Table 4. Hazard ratios (HRs) and 95% confidence intervals (CIs) for the effect of social isolation and loneliness on all-cause mortality

	All<br>HR (95% CI)	Women<br>HR (95% CI)	Men<br>HR (95% CI)
Social isolation			
0 point	Reference	Reference	Reference
1 point	1.36 (1.31-1.41)	1.32 (1.24-1.40)	1.39 (1.32-1.46)
2 point	2.01 (1.90-2.13)	1.88 (1.70-2.05)	2.12 (1.97-2.27)
3 point	3.38 (2.92-3.83)	2.71 (1.91-3.51)	3.65 (3.09-4.20)
Loneliness			
0 point	Reference	Reference	Reference
1 point	1.25 (1.20-1.30)	1.22 (1.14-1.30)	1.28 (1.21-1.34)
2 point	1.59 (1.48-1.69)	1.44 (1.28-1.59)	1.71 (1.56-1.85)

Analyses are adjusted for age, sex, and ethnicity.

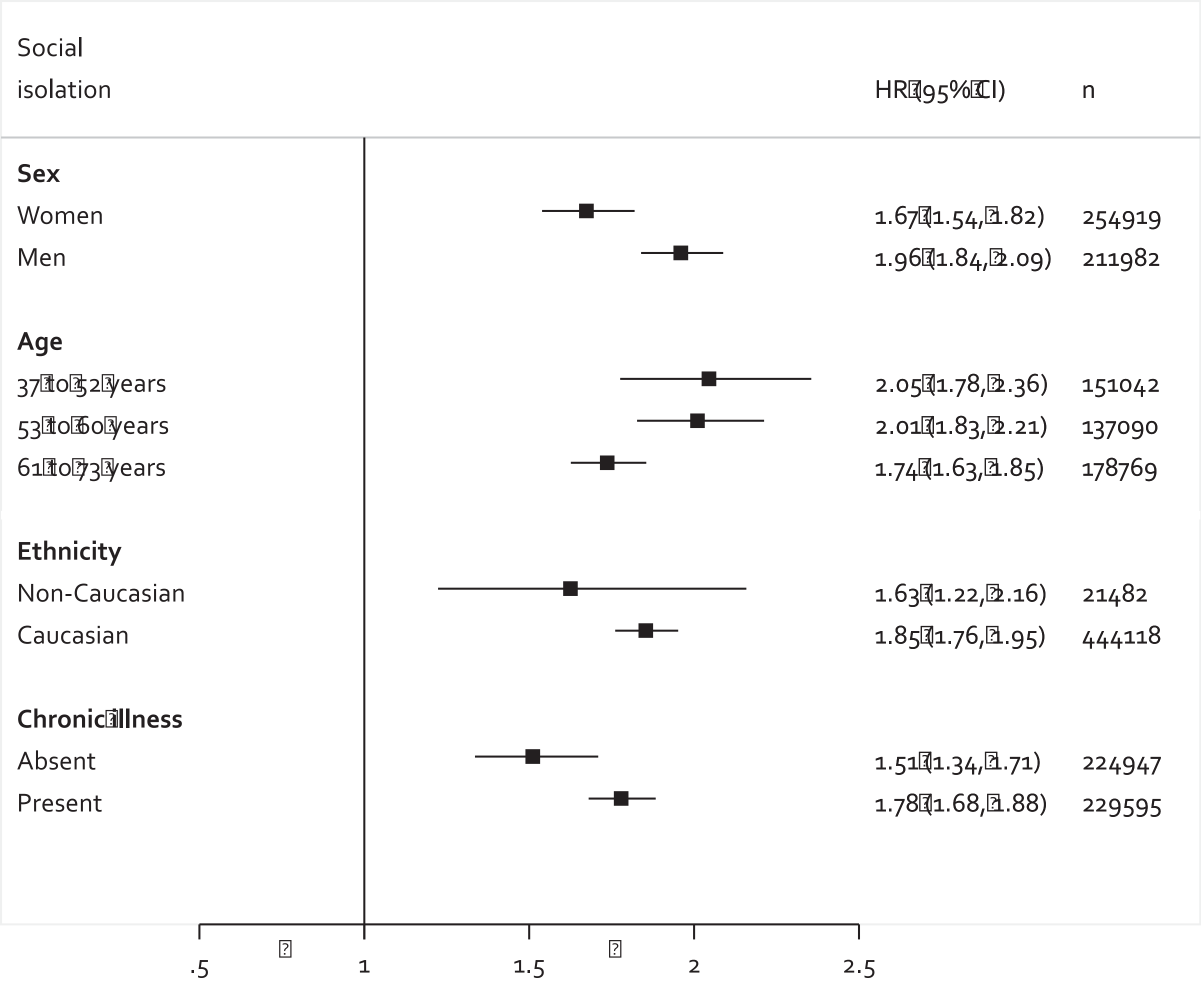

Supplement figure 1. Hazard ratios (HRs) for the association of social isolation (compared to no social isolation) with all-cause mortality in various sub-groups of potential baseline confounders

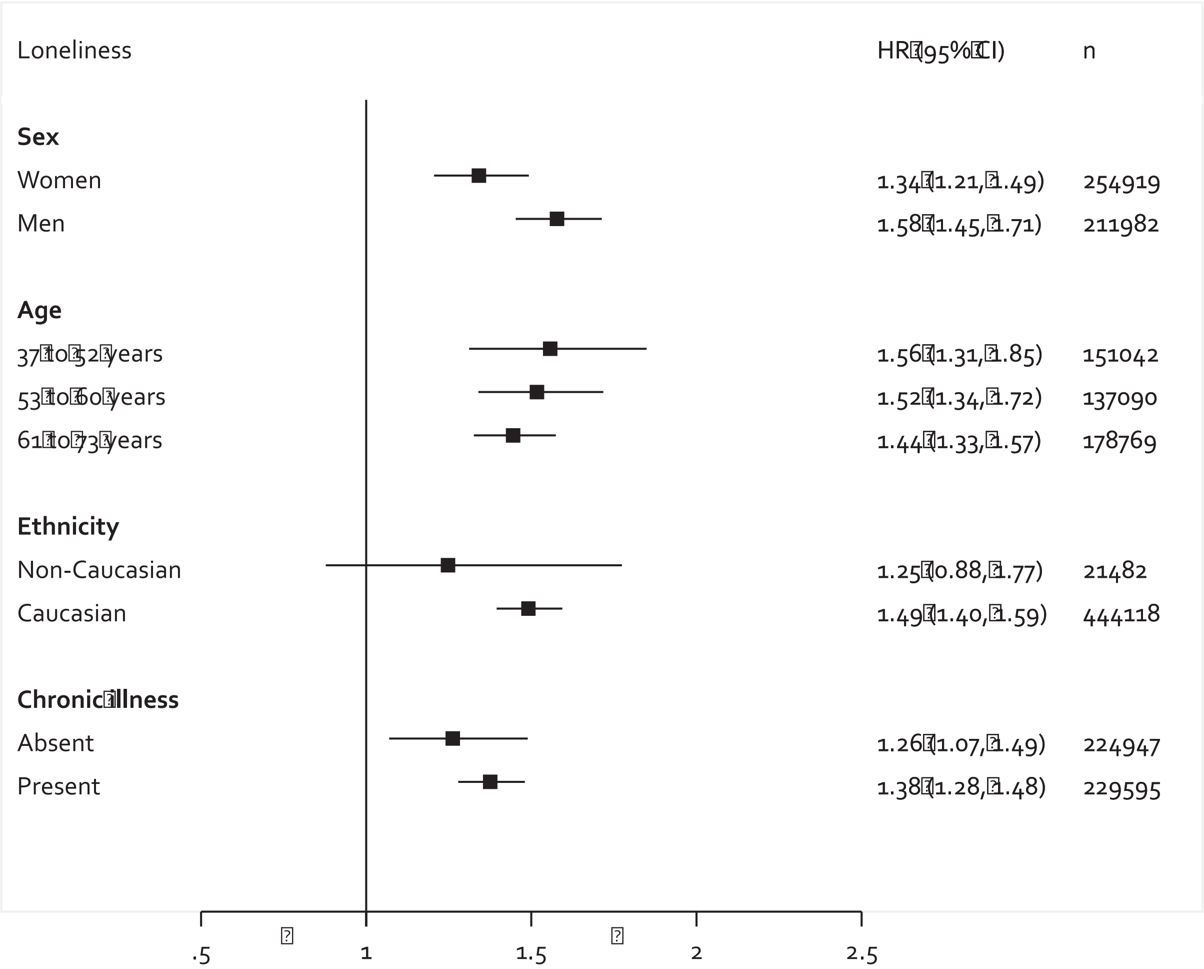

Supplement figure 2. Hazard ratios (HRs) for the association of loneliness (compared to no loneliness) with all-cause mortality in various sub-groups of potential baseline confounders
